# Lax eyelid condition (LEC) and floppy eyelid syndrome (FES) prevalence in obstructive sleep apnea syndrome (OSA) patients: a systematic review and meta-analysis

**DOI:** 10.1007/s00417-022-05890-5

**Published:** 2022-11-16

**Authors:** Francesco Aiello, Gabriele Gallo Afflitto, Mario Alessandri Bonetti, Francesca Ceccarelli, Massimo Cesareo, Carlo Nucci

**Affiliations:** 1 Ophthalmology Unit, Department of Experimental Medicine, University of Rome “Tor Vergata”, Rome, Italy; 2 Bascom Palmer Eye Institute, University of Miami Miller School of Medicine, Miami, FL 33136 USA; 3 Department of Reconstructive and Aesthetic Plastic Surgery, University of Milan, I.R.C.C.S. Istituto Galeazzi, Milan, Italy

**Keywords:** Lax eyelid condition, LEC, Floppy eyelid syndrome, FES, Obstructive sleep apnea syndrome, OSA, Epidemiology

## Abstract

**Purpose:**

Lax eyelid condition (LEC) and floppy eyelid syndrome (FES) represent two distinct conditions which have been associated with several ocular and systemic comorbidities. The main aim of this systematic review and meta-analysis is to explore the available literature to estimate the prevalence rate of LEC and FES in obstructive sleep apnea (OSA).

**Methods:**

The protocol of this systematic review and meta-analysis has been registered in PROSPERO. Four electronic databases (PubMed/MEDLINE, Google Scholar, Cochrane Library, Web of Science) were searched from inception to December 24, 2021. A random intercept logistic regression model was carried out for the analysis of overall proportions. Odds ratio and mean difference were reported as measures of the effect size in the presence of binary and continuous outcomes, respectively. The estimated numbers of LEC/FES patients in OSA were calculated by multiplying the prevalence rate determined by our random-effects model and the corresponding Benjafield et al.’s population prospect.

**Results:**

We included 11 studies comprising 1225 OSA patients of whom 431 and 153 affected by LEC and FES, respectively. Our model estimated a pooled prevalence rate for LEC and FES in OSA patients of 40.2% (95%CI: 28.6–53.1%) and of 22.4% (95%CI: 13.8–34.2%), respectively. The number of LEC/FES affected individuals among OSA patients is expected to peak up to 376 and to 210 million, respectively. OSA patients appeared to have a 3.4 (95%CI: 2.2–5.2) and a 3.0 (95%CI: 1.7–5.5) increased risk of developing LEC and FES than the healthy counterpart.

**Conclusion:**

Prevalence of LEC and FES is higher in OSA-affected patients compared to controls. More studies are warranted to investigate the mechanisms leading to the development of LEC and/or FES in OSA patients, as well as the feasibility of the adoption of these clinical findings as screening tools for OSA.

**Supplementary Information:**

The online version contains supplementary material available at 10.1007/s00417-022-05890-5.

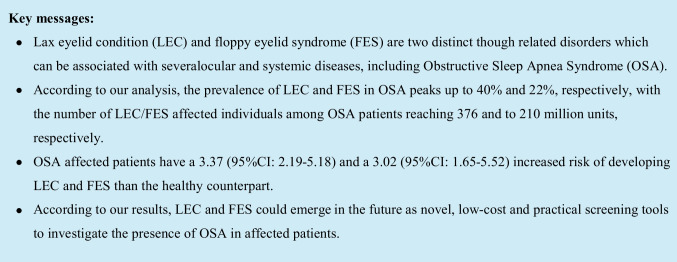



## Introduction

“Lax eyelid condition” (LEC) refers to the presence of a thin, rubbery, and easily eversible upper tarsus, whereas “floppy eyelid syndrome” (FES) is recognized by the evidence of the macroscopic features of the LEC in association with a chronic papillary conjunctival response in the upper lid [1–3]. Similarities in their macroscopic presentation make of LEC and FES two related clinical entities [1, 3].

In the last two decades, a growing number of studies aimed at better elucidating the clinical and epidemiological features of both LEC and FES [4–6]. A plethora of different ocular (i.e., open angle glaucoma, normo-tensive glaucoma, ischemic optic neuropathy) [7, 8] and systemic disorders (i.e., obesity, hypertension, obstructive sleep apnea syndrome) emerged as eventually occurring with LEC and FES [1, 2, 6].

Obstructive sleep apnea syndrome (OSA) is characterized by recurrent episodes of partial or complete collapse of the upper airway during sleep, responsible for intermittent hypoxemia and sleep fragmentation [9, 10]. Several risk factors have been identified for OSA (e.g., high BMI, sex, postmenopausal state, enlarged upper airway soft tissues, craniofacial abnormalities). Notably, this condition affects nearly 20–25% of the adult population (> 18 years) in the USA, but it might be encountered too in 1.2 to 5.7% of the pediatric population [9–11]. The screening methodologies nowadays available for OSA consist of specific questionnaires (e.g., Berlin Questionnaire, STOP-Bang questionnaire), while polysomnography is the current gold standard diagnostic test for sleep apnea [11, 12]. Interestingly, no physical examination findings specific to OSA have been identified, which might serve as useful tools to identify at-risk individuals [12].

In 1990, Woog et al. suggested an association between FES and OSA, which has since been further investigated, leading to contradictory results [2, 13, 14]. Specifically, two meta-analyses explored the epidemiological link existing between LEC/FES and OSA, reporting a nearly fourfold increased risk of FES/LEC in OSA [15, 16]. However, both works failed to distinguish the nosological differences existing between FES and LEC, being data of patients with LEC and FES mixed in the pooled analysis. In addition, none of those studies tried to ascertain the epidemiological burden of FES and LEC, so that a reliable estimate of the total number of LEC- and FES-affected individuals among OSA patients is not yet available in the literature. Furthermore, not having any previous meta-analysis tried to separately analyze the prevalence of LEC and FES in OSA, a question remains regarding the existence of any possible associations between these two different entities in sleep apnea patients. This is especially true considering the potential blinding complications of FES, not reported in the context of LEC [8].

The main aim of this systematic review and meta-analysis is to explore the literature to estimate the prevalence rate of LEC and FES in OSA. Our analysis will use the latest available data to both emphasize the differences in prevalence between LEC and FES in OSA and to estimate the total number of LEC/FES-affected individuals among OSA patients.

## Materials and methods

This study was reported in accordance with the Preferred Reporting Items for Systematic Reviews and Meta-Analysis (PRISMA) guidelines [17]. Since all the presented data were obtained from the available published literature, neither institutional review board approval nor informed consents were required for the completion of this study. The study protocol of this systematic review and meta-analysis was sent, scrutinized, approved, and registered in the international prospective register of systematic reviews (PROSPERO) (CRD42022302588).

### Inclusion and exclusion criteria

The PICOS framework [18] was used in developing the literature search strategy, therefore including *patients* (P), male and female adults worldwide (> 18 years) affected by OSA; *investigated condition* (I), LEC classified according to both the Beis et al. [19] and the Acar et al. [20] descriptions, and FES defined by the presence of eyelid hyperlaxity associated with chronic papillary conjunctivitis; *comparator* (C), OSA unaffected patients; *outcome* (O), prevalence rate; *study type* (S), prospective and retrospective observational cohort studies. Notably, studies were excluded if they (a) were not in English; (b) were in the form of either a conference abstract, a review, a case report, a book chapter or a letter to the editor; (c) included < 70% of patient assessments directly performed by the investigators; and (d) were not available in full text form.

### Data source and study searching

An electronic search was performed on PubMed/MEDLINE, Google Scholar, Cochrane Library, and Web of Science using relevant keywords, phrases, and medical subject headings (MeSH) terms, from inception to 24 December 2021 (date of last search). It is worth noting that no articles would have been included in our analysis with the last database search being conducted on August 20, 2022 (date of peer-review). The search strings applied for different databases are reported in supplementary material. We applied the “cited by” tool on Google Scholar to minimize the risk of missing relevant works. The reference list of each selected article was then checked to screen for additional relevant studies, as per the snowballing method.

### Data extraction

Two reviewers independently conducted the electronic literature search (F.C., M.A.B.). The reference lists from the 4 databases (i.e., PubMed/MEDLINE, Google Scholar, Cochrane Library, Web of Science) were merged and the duplicates removed using the reference management software EndNote X9 (version X9.3.3). After title and abstract screening, the full text of remaining papers was analyzed. In the presence of eventual discrepancies in the selection process, a third reviewer (F.A.) was consulted to solve the conundrum. Two reviewers (F.C., M.A.B.) collected relevant data from included reports. No automation tools were used in the process. The following variables were extracted by each included manuscript: author and year of publication; country of origin; total number of screened subjects; number of LEC- and/or FES-affected patients in the study group; total number of OSA affected patients; number of LEC- and/or FES-affected patients in the control group; total number of healthy subjects; corresponding demographic features including age, BMI, and severity of OSA according to the apnea-hypopnea index [10].

Data extracted from selected papers were archived in a customized Excel (Microsoft Corp, Seattle, Washington, USA) spreadsheet with forced choice entry criteria. Corresponding authors of related articles were contacted to obtain missing data. Whenever relevant data was not available, the corresponding study was excluded by the pooled analysis for that endpoint.

### Risk of bias and study quality assessment

Two senior reviewers (F.A., M.C.,) independently evaluated the quality of the included studies according to the Joanna Briggs Institute Prevalence Critical Appraisal Tool (JBI-PCAT) [21]. As recently proposed by the Prevalence Estimates Reviews — Systematic Review Methodology Group (PERSyst), the JBI-PCAT represents the most appropriate tool to assess the methodological quality of prevalence studies [22]. Reporting bias was assessed in accordance with the JBI-PCAT.

### Data synthesis and statistical analysis

The analysis was performed using the R software for statistical computing (R 1.4.1106; “meta” package). Due to the high level of expected heterogeneity, the random effects model was used, whose results are presented with forest plot graphs.

The main aim of this systematic review and meta-analysis was to compare the prevalence of LEC-and FES-affected individuals among OSA patients. Hence, two separate analyses for LEC and FES were conducted per each studied variable.

Logit transformation (PLOGIT) of data and a random intercept logistic regression model (GLMM) were carried out for the analysis of overall proportions, which were expressed in association with a 95% Clopper-Pearson confidence interval. Mean difference was calculated as a measure of effect size to compare continuous variables and expressed with 95% Clopper-Pearson confidence interval. Odds ratio (OR) and the relative 95% Clopper-Pearson confidence interval were calculated using the “metabin” function of the “meta” package in R to compare binary outcomes. Statistical significance was defined as *p* < 0.05.

Cochran’s Q was calculated as a measure of heterogeneity and checked by *p-*value. We also reported *I*^2^ statistic results, which quantify heterogeneity regardless of the number of included studies. The maximum-likelihood estimator was used to estimate the between-study variance (*τ*^2^).

Influence analysis was performed using the “InfluenceAnalysis” function in R, and a Baujat plot was consequently created. Outliers analysis was conducted using the “find.outliers” function in R.

The risk of publication bias was quantitatively assessed by the Peters’ linear regression test. A contour enhanced funnel plot was created as an aid to differentiating asymmetry due to publication bias from that due to other factors.

The estimated population of OSA-affected patients was retrieved from the Benjafield et al.’s analysis, which represents the first report on the global prevalence of OSA, diagnosed according to both the American Academy of Sleep Medicine 2012 Scoring Criteria and to the equivalent Apnea-Hypopnea Index (AHI) [10]. The estimated numbers of LEC/FES patients were calculated by multiplying the prevalence rate determined by our random-effects model and the corresponding Benjafield et al.’s population prospect.

## Results

### Electronic Database Search results

**Fig. 1:**
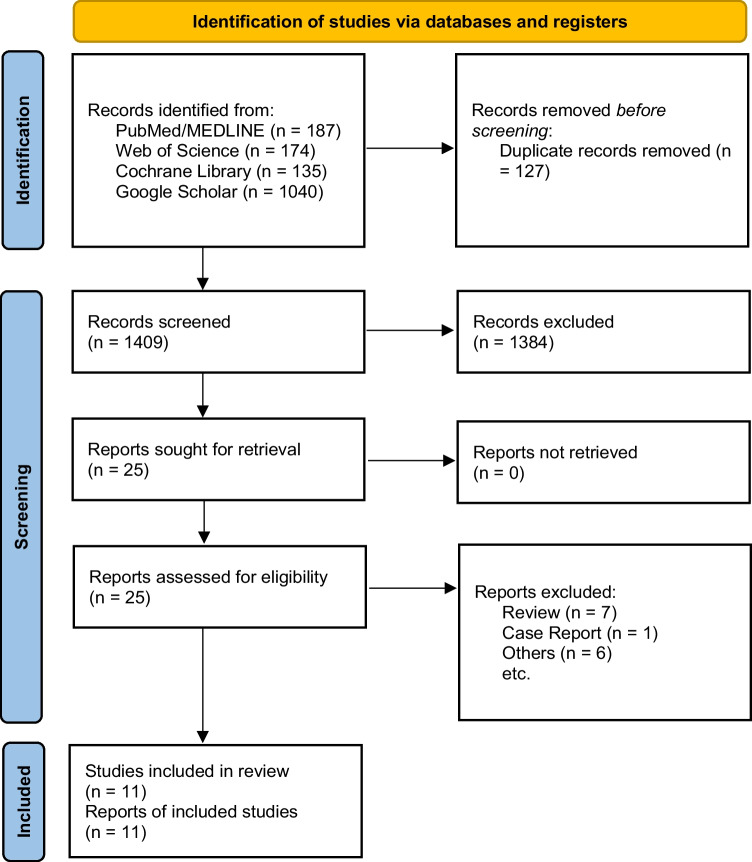
Preferred reporting items for systematic review and meta-analysis flowchart. Reasons for exclusion are step-by-step reported on the right.

A total of 1536 eligible papers (i.e., 187 from PubMed/MedLine, 174 from Web of Science, 135 from Cochrane Library, and 1040 from Google Scholar) were retrieved from the preliminary search on electronic databases. After automatic duplicates removal and both screening of titles and abstracts, 25 full-text manuscripts were assessed for eligibility. Globally, 11 articles were included in the qualitative and quantitative analysis (Fig. 1), whose general features are reported in supplementary materials [19, 20, 23–31]. Specifically, 9/11 [19, 20, 23, 24, 26–29, 31] and 7/11 [19, 23, 25–27, 29, 30] studies reported information regarding the prevalence of LEC and FES among OSA-affected patients respectively. Five out of 11 articles were found to describe both LEC and FES distribution in OSA [19, 23, 26, 27, 29]. Additionally, 2 [20, 27] and 3 [26, 27, 30] studies reported information about age, BMI, and prevalence of LEC and FES respectively, according to OSA severity.

Participants took part to the studies from 1996 to 2020, and articles were published between 1997 and 2020. All studies were either cross-sectional or case-control and hospital-based, and data were collected prospectively in the totality of cases (100%).

Eight (72.7%) of the 11 studies had a moderate risk of bias as evaluated by the JBI-PCAT tool (view supplementary materials) [19, 20, 24, 25, 27–29, 31]. One only study demonstrated a high risk of bias (3/9 JBI-PCAT items), featuring unclear randomization and data-handling protocols, and an inconsistent description of the recruited sample and of the applied statistical methods [23].

### Lax eyelid condition in OSA-affected patients

Overall, 431 patients in our sample were found to present with LEC (i.e., 384 OSA; 47 non-OSA). The pooled mean age of the OSA-affected group assuming a random-effect model was 54.7 years old (yo) (95%CI: 50.1-59.4 yo), significantly different from the one of the control group, which was evaluated as being 47.6 yo (95%CI: 43.8–51.4 yo) (*p* = 0.0263) (view supplementary materials). Similarly, the pooled mean BMI of the OSA- and non-OSA groups assuming a random-effects model was 32.4 kg/m^2^ (95%CI: 29.9–34.7 kg/m^2^) and 29.4 kg/m^2^ (95%CI: 26.4–32.5 kg/m^2^), respectively (*p* < 0.0001) (view supplementary materials).

**Fig. 2:**
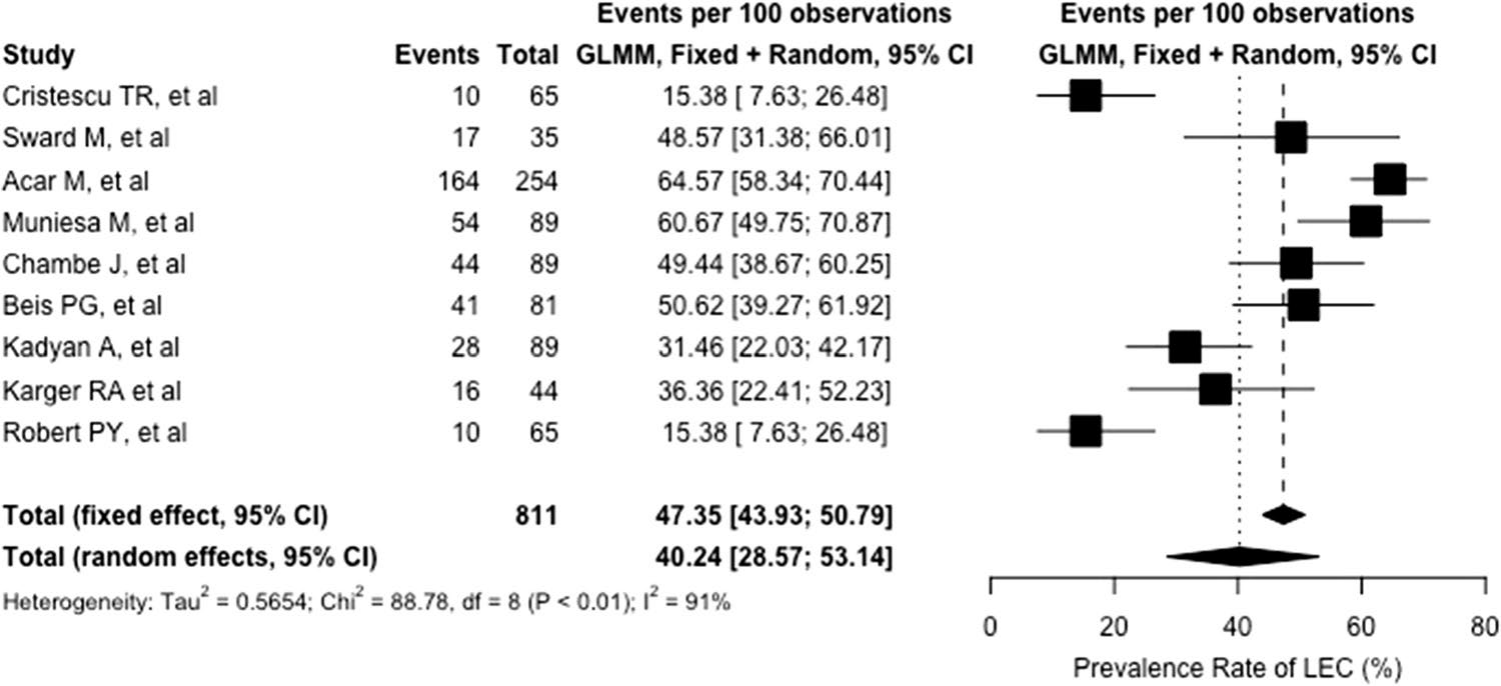
Forest plot showing the single and pooled estimate of lax eyelid condition prevalence rate. Both fixed and random effect models are represented. (GLMM: generalized linear mixed model; LEC: lax eyelid condition)

Globally, the pooled prevalence rate of LEC, as determined by our random-effect model, was determined to be 40.2% (95%CI: 28.6–53.1%). The heterogeneity variance among different studies was estimated at *τ*^2^ = 0.5654, with an *I*^2^ value of 91.0% (95%CI: 85.2–94.5%). Pooled results are reported in the forest plot presented in Fig. 2. Results deriving from the Baujat plot and the influence and sensitivity analysis are reported in supplementary materials. Interestingly, both the outlier and the influence analysis revealed the Cristescu et al., the Acar et al., and the Robert studies to majorly impact the overall heterogeneity of our results.

The funnel plot generated is shown in supplementary materials. It shows a moderate asymmetry, though Peter’s test results did not reveal apparent publication bias (*t* = − 1.77, *p* = 0.1197).

**Fig. 3:**
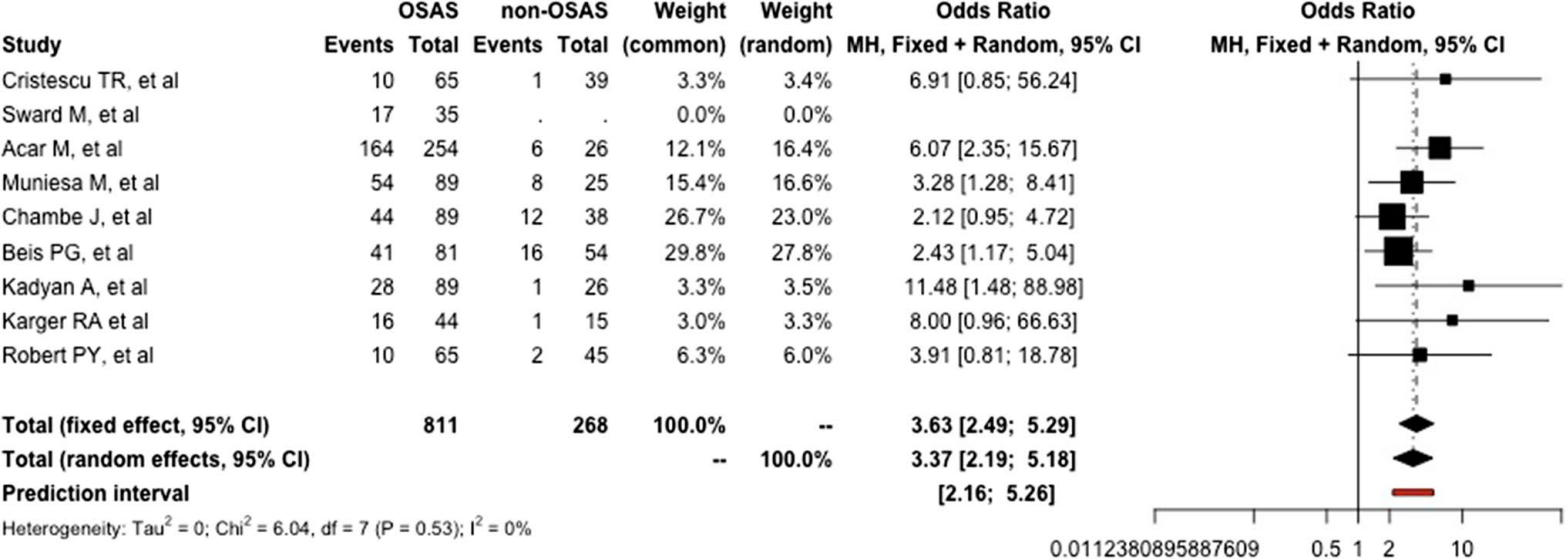
Forest plot resuming the single and pooled odds ratio of lax eyelid condition according to the presence of obstructive sleep apnea syndrome. (GLMM: generalized linear mixed model)

Overall, according to our randomized effect model, OSA-affected patients appeared to have a 3.4 (95%CI: 2.2-5.2; *I*^2^ = .0%) increased risk of developing LEC than the non-OSA counterpart (*p* = 0.0003) (Fig. 3).

Additional features of LEC-affected patients according to the severity of OSA are reported in Table 1. No meta-regression was performed due to the number of included studies being < 10.

**Table 1:** Characteristics of lax eyelid condition–affected patients according to obstructive sleep apnea syndrome severity. Odds ratios (95%CI) are calculated with respect to healthy control (i.e., non-OSA individuals). (OSA: obstructive sleep apnea syndrome; LEC: lax eyelid condition; BMI: body mass index; CI: confidence interval; NA: not available)

	Non-OSAS	Mild OSAS	Moderate OSAS	Severe OSAS
Age, mean (95%CI)	45.63 (43.26-48.00)	43.90 (40.99–46.81)	49.90 (47.75–52.05)	58.11 (57.53–58.70)
BMI, mean (95%CI)	26.84 (10.45–43.23)	29.50 (28.23–30.77)	31.50 (30.16–32.84)	30.30 (29.93–30.68)
LEC (OR, 95% CI)	NA	2.38 (0.84–6.78)	6.67 (2.34–18.78)	8.30 (3.84–17.97)

### Floppy eyelid syndrome in OSA-affected patients

The total number of FES-affected patients in this cohort peaked up at 153 (i.e., 133 OSA; 20 non-OSA). Statistically significant differences emerged from the comparison of age (view supplementary materials) and BMI (view supplementary materials) between the study and the control group (OSA age: 56.8 yo (95%CI: 51.1–62.4 yo); non-OSA age, 39.2 yo (95%CI: 21.4–56.9 yo); *p* = 0.0428) (OSA BMI: 32.3 kg/m^2^ (30.1–34.5 kg/m^2^); non-OSA BMI, 29.1 kg/m^2^ (95%CI: 26.5; 31.7 kg/m^2^); *p* = 0.0057).

**Fig. 4:**
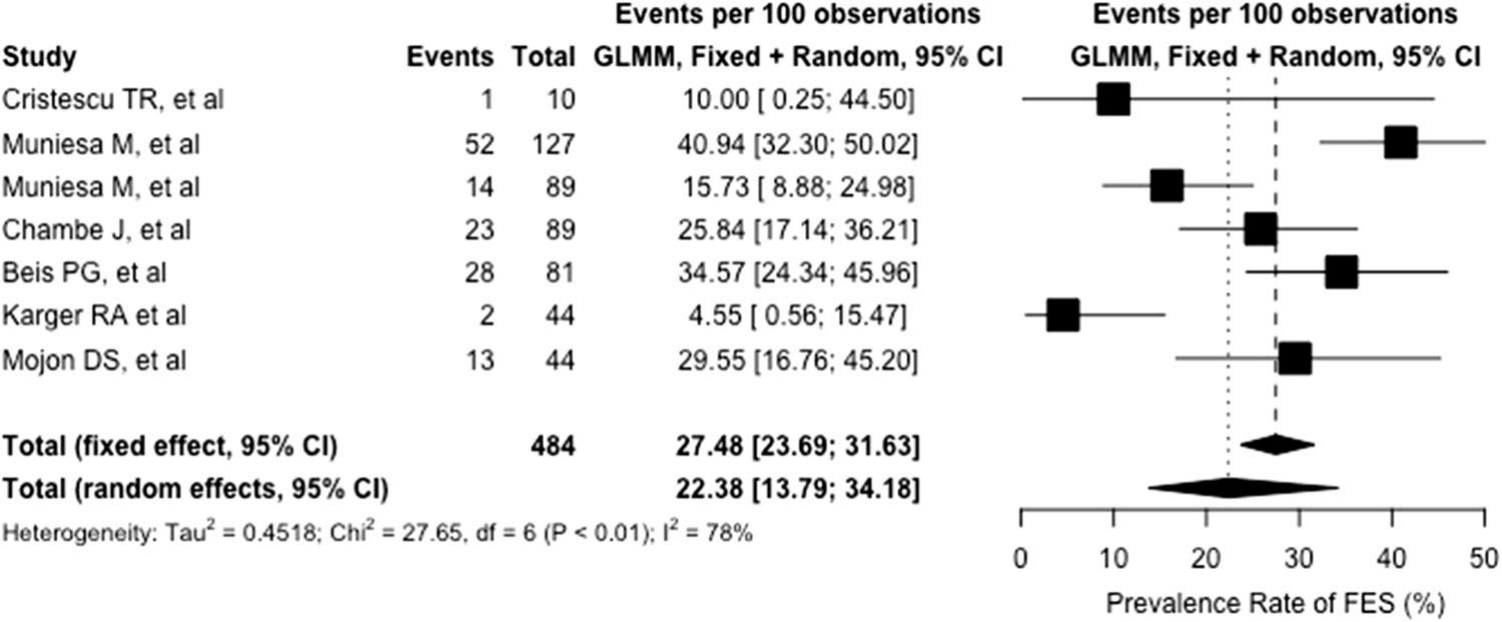
Forest plot showing the single and pooled estimate of floppy eyelid syndrome prevalence rate. Both fixed and random effect models are represented. (GLMM: generalized linear mixed model; FES: floppy eyelid syndrome)

According to our random-effect model, the global prevalence of FES among OSA-affected patients was 22.4% (95%CI: 13.8–34.2%). Pooled results are summarized in the forest plot in Fig. 4. The heterogeneity variance among different studies was estimated at *τ*^2^ = 0.4518, with an *I*^2^ value of 78.1% (95%CI: 55.1–89.5%). While no outliers were found, the influence analysis function revealed data from Karger et al. to majorly impact on the heterogeneity of pooled results, as shown in supplementary materials.

Only a partial asymmetry emerged from the analysis of the funnel plot (view supplementary materials), with the Peter’s test not apparently suggesting any publication bias (*t* = − 1.01, *p* = 0.3568).

**Fig. 5:**
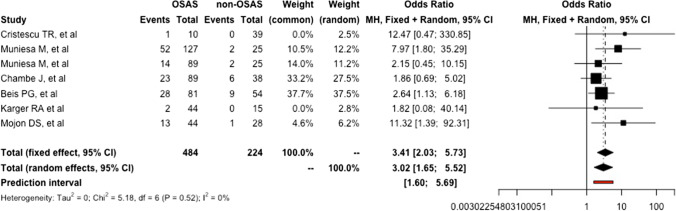
Forest plot resuming the single and pooled odds ratio of floppy eyelid syndrome according to the presence of obstructive sleep apnea syndrome. (GLMM: generalized linear mixed model)

Nonetheless, our random-effect model demonstrated a 3.0 (95%CI: 1.65–5.52; *I*^2^ = 0%) increased risk of developing FES in OSA-affected patients than in healthy controls (*p* = 0.0042) (Fig. 5).

Additional features of the FES-affected patients according to OSA severity are summarized in Table 2.

**Table 2:** Characteristics of floppy eyelid syndrome-affected patients according to obstructive sleep apnea syndrome severity. Odds ratios (95%CI) are calculated with respect to healthy control (i.e., non-OSA individuals). (OSA: obstructive sleep apnea syndrome; LEC: lax eyelid condition; BMI: body mass index; CI: confidence interval; NA: not available)

	Non-OSAS	Mild OSAS	Moderate OSAS	Severe OSAS
Age, mean (95%CI)	45.70 (45.10–46.29)	51.10 (45.09–57.11)	62.38 (61.54–63.22)	58.11 (57.53–58.69)
BMI, mean (95%CI)	25.78 (25.56–25.99)	29.60 (27.18–32.01)	33.27 (31.37–35.15)	30.43 (30.05–30.81)
FES (OR, 95% CI)	NA	3.24 (0.60–17.41)	2.64 (0.62–11.29)	4.68 (1.94–11.25)

No meta-regression was performed due to the number of included studies being < 10.

### Number of OSA-affected patients with LEC or FES worldwide

According to Benjafield et al.’s population prospect of OSA-affected patients in 2019 (i.e., 936 million), and to the prevalence estimates derived from our GLMM, the total number of LEC and FES individuals peaks up to 376 and to 210 million, respectively. As per the paucity of the available data, no age-, gender-, or geographic-related differences in the LEC-FES population prospects were investigated.

## Discussion

The main aim of the present work was to distinctly ascertain the prevalence of LEC and FES in patients affected by OSA. An estimate of the global epidemiological burden of the 2 different conditions in such a selected population was provided too, as being of 376 and of 210 million individuals, respectively.

Despite several attempts to establish its clinical significance [3, 32], the term floppy eyelid syndrome has been inconsistently used as a proxy for several clinical scenarios in which eyelid laxity, with or without anterior segment pathology, was observed. Among them, laxity due to habitual eyelid manipulation, congenital deformities, inheritable hyperlaxity conditions, hyperglycinemia, and other mechanical or tissue pathology has been included [32–36]. However, while phenotypically similar, no data are nowadays available demonstrating an etiological link between LEC and FES [2]. In addition, the total number of LEC-affected subjects overcomes the one of patients with FES, assuming the latter as representing a specific subgroup of patients with LEC. Hence, a different epidemiological burden of the two conditions must be considered, as demonstrated by our results.

According to the analysis of the data retrieved by the second phase of the Shahroud Eye Cohort Study (ShECS), 11.3% of the population aged 45 to 69 years had FES [4]. This estimate appears to be substantially contracted when compared to the one of 40.2% and of 22.4% we reported for LEC and FES respectively. However, while we restricted our observation to OSA-affected patients for the purposes of the present study, the ShECS represents a large epidemiological study applying a random cluster sampling of the general population as the main selection criteria [4].

Of note, two other meta-analyses from Wang et al. and Huon et al. have beforehand reported a statistically significant increased odd for OSA-affected individuals to develop FES compared to healthy individuals (i.e., 4.1 and 4.7) [15, 16]. Though in line with these results, our data appears to be slightly undersized, as we described only a trifold odd for LEC and FES in the study group than in the control one. Several reasons might potentially explain this discrepancy but, to our opinion, selection criteria applied in our meta-analysis are the first and foremost one. In fact, as previously mentioned, we decided to clearly distinguish LEC and FES as two different disease entities, featured by a specific set of anatomic and physiologic findings, coherently to what was described by Fowler et al. [2]. As a matter of fact, the pooled analysis from both Wang et al. and Huon et al. did not take into account any eventual difference between LEC and FES. An oversizing thus resulted mainly due to the majorly prevalent LEC. Nonetheless, while we preferred to refer to a randomized GLMM, odds ratios by Wang et al. were pooled according to a fixed-effect model [16].

Our randomized models, globally including 1225 patients, were sufficiently powered to detect statistically significant differences between the study and the control group in terms of age and BMI, as extensively reported in the literature [9]. While this evidence further corroborates the reliability of our findings, it is interesting to note how the measured BMI in the control group bordered on the obesity cut-off [37]. While discouraging, this data appears to be in line with the data reported in European Countries by the Eurostat indicating 51.3% overweight individuals out of the entire European population (i.e., we referred to European projections being the majority of the included studies Europe-based) [38]. Due to the paucity of included studies, we were unable to run any meta-regression to explore both BMI and age as predictors of the pooled effect size, which have been reported to significantly correlate to LEC and FES development in multivariable regression models [4].

Furthermore, a progressively increasing odd for both LEC and FES was registered according to the OSA severity, here defined. While this data complies with that reported by Wang et al. [16], it is noteworthy the exiguous number of studies included in the sub-analysis, which invariably affect the validity of the proposed results.

Intriguingly, all these results seem to substantiate one of the main pathophysiological theories not only explaining the occurrence of LEC and FES, but also justifying their frequent overlap with the OSA. In fact, it has been proposed that the high BMI, typical of OSA patients, associates with elevated plasma leptin concentration [39]. Interestingly, leptin and ischemia-reperfusion injury (i.e., typical of sleep apnea) have been shown to regulate the expression of matrix metalloproteinase-9 (MMP-9) [2, 40]. Increased expression of MMP-9 and depleted elastin in the tarsal connective tissue, as well as a loss of elastic fibers in the eyelid skin and around the ciliary roots, were found in patients with LEC [41]. Additionally, it has been proposed that the loss of tarsal elasticity leads to nocturnal eyelid eversion which in turn could cause a chronic papillary response in the upper tarsus, representative feature of FES.

However, while intriguing, this speculation is unable to explain some other observations on subjects. As a fact, it is unclear why only few patients with OSA manifest a full constellation of signs and symptoms of LEC or FES, with the prevalence of OSA among FES/LEC patients ranges between 2 and 5% [5, 42]. In addition, assuming irreversible ultrastructural changes in the extracellular matrix induced by sleep apnea, it is not clear how continuous positive airway pressure (CPAP) treatment might help to ameliorate FES/LEC symptoms overtime in OSA patient [5, 42]. Nonetheless, ocular irritation from CPAP has been reported, too, further complicating the scenario [28].

Among the strengths of our meta-analysis, the critical appraisal of study quality, the rigorous application of diagnostic criteria, and the strict observation of inclusion and exclusion criteria must be considered. In addition, subgroup analyses, influence analyses, and, finally, a sensitivity analysis were run to ensure the robustness of results. As for every meta-analysis, our study too has some limitations [43–45]. Primary studies included in this work were conducted only in an exiguous number of countries, making the reported estimates of LEC and FES prevalence in OSA patients not fully representative. Furthermore, because all the analyzed data derived from studies conducted in tertiary hospitals, our findings might not be generalizable to lower-level health-care facilities. We found a substantial heterogeneity in most analyses. This heterogeneity can be partly explained by modifiable and unmodifiable external factors, including different demographic features of the included populations, case definition, diagnostic criteria applied, and diverse human and infrastructural resources across studies.

As our results suggested LEC having nearly double the prevalence of FES in OSA patients, the sharp distinction between the two entities we delineated was shown to be meaningful. On one hand, our data proposes that not only FES but also LEC might be considered suggestive of OSA. On the other hand, it should be acknowledged that, while less common, FES has been associated with serious and potentially blinding corneal complications [8]. As reported by Din et al., in a retrospective 10-year consecutive case-series, 6.73% of FES diagnosed patients developed severe chronic keratitis, for whom both medical and surgical corneal treatments were required [8]. Clinical deterioration of affected patients resulted as a consequence of a delay in the diagnosis of FES, the author concluded. Hence, the importance of discriminating between FES and LEC, especially considering that similar events have not yet been reported in the context of LEC.

The relative high frequency of both LEC and FES in OSA-affected patients already proposed by the literature is corroborated by our findings. Though a direct evaluation of the positive predictive diagnostic performance of LEC or FES with regard to OSA was not feasible, our findings promote LEC and FES as possible indices of sleep apnea [5].

In this context, pathophysiological studies are warranted to better elucidate the mechanisms leading to the development of LEC and/or FES in OSA patients. In addition, large prospective population studies are needed to further explore the relation existing between LEC/FES and OSA, to properly evaluate the natural history of such conditions in the context of sleep apnea, the impact of OSA severity in their pathogenesis, and the feasibility of the adoption of these clinical findings as screening tools for OSA. The latter appears to us a crucial main point of analysis if referred to a condition having a notable economic and psychological impact on the affected population and which remains undetected in the vast majority of cases, as OSA is.

## Supplementary Information

Supplementary file 1 **S0**. Search query used in different databases. (DOCX 13 kb)

Supplementary file 2 **S1**. General Features of the included studies. (Abbreviations: SD - Standard deviation -; Geo - geographical localization of the study -; RoB - Risk of Bias -; LEC – Lax Eyelid Condition -; FES – Floppy Eyelid Syndrome -; NA – Not Available -.) (DOCX 15 kb)

Supplementary file 3 **S2**. Risk of bias assessment according to the Joanna Briggs Institute Prevalence Critical Appraisal Tool. (a. Was the sample frame appropriate to address the target population?; b. Were study participants recruited in an appropriate way?; c. Was the sample size adequate?; d. Were the study subjects and setting described in detail?; e. Was data analysis conducted with sufficient coverage of the identified sample?; f. Were valid methods used for the identification of the condition?; g. Was the condition measured in a standard, reliable way for all participants?; h. Was there appropriate statistical analysis?; i. Was the response rate adequate, and if not, was the low response rate managed appropriately?) (DOCX 15 kb)

Supplementary file 4 **S3.** Forest plot showing mean difference of age between the experimental and the control group in the included studies analyzing the prevalence of lax eyelid condition. (PNG 948 kb)

High Resolution Image (TIFF 2639 kb)

Supplementary file 5 **S4.** Forest plot showing mean difference of body mass index between the experimental and the control group in the included studies analyzing the prevalence of lax eyelid condition. (PNG 902 kb)

High Resolution Image (TIFF 2639 kb)

Supplementary file 6 **S5.** Baujat plot of the studies analyzing the prevalence rate of lax eyelid condition. (PNG 329 kb)

High Resolution Image (TIFF 1253 kb)

Supplementary file 7 **S6.** Influence analysis of the studies analyzing the prevalence rate of lax eyelid condition. (PNG 939 kb)

High Resolution Image (TIFF 1253 kb)

Supplementary file 8 **S7.** Sensitivity analysis conducted in accordance with the run influence analysis. (LEC: Lax Eyelid Condition; CI: Confidence interval; OSA: obstructive sleep apnea syndrome) (DOCX 12 kb)

Supplementary file 9 **S8.** Contour enhanced funnel plot of the studies analyzing the prevalence rate of lax eyelid condition. (LEC: Lax Eyelid Condition) (PNG 362 kb)

High Resolution Image (TIFF 1106 kb)

Supplementary file 10 **S9.** Baujat plot of the studies analyzing the prevalence rate of floppy eyelid syndrome. (PNG 310 kb)

High Resolution Image (TIFF 1106 kb)

Supplementary file 11 **S10.** Influence analysis of the studies analyzing the prevalence rate of floppy eyelid syndrome. (PNG 883 kb)

High Resolution Image (TIFF 1106 kb)

Supplementary file 12 **S11.** Sensitivity analysis conducted in accordance with the run influence analysis. (FES: Floppy Eyelid Syndrome; CI: Confidence interval; OSA: obstructive sleep apnea syndrome) (DOCX 14 kb)

Supplementary file 13 **S12.** Contour enhanced funnel plot of the studies analyzing the prevalence rate of floppy eyelid syndrome. (FES: Floppy Eyelid Syndrome) (PNG 358 kb)

High Resolution Image (TIFF 1106 kb)

## Data Availability

The data that support the findings of this study are available from the corresponding author upon reasonable request.
