# Supplementary material for: Lax eyelid condition (LEC) and floppy eyelid syndrome (FES) prevalence in obstructive sleep apnea syndrome (OSA) patients: a systematic review and meta-analysis
Source: Graefes Arch Clin Exp Ophthalmol. 2022 Nov 16;261(6):1505–14. doi: 10.1007/s00417-022-05890-5 (PMC10198907; doi:10.1007/s00417-022-05890-5)
Supplement: Supplementary file 1 — S0. Search query used in different databases. (DOCX 13 kb) [file 417_2022_5890_MOESM1_ESM.docx]

| **Database** | **Search String** | **N°of Results** |
| --- | --- | --- |
| PubMed/MEDLINE | ((((((((floppy eyelid)[Title/Abstract])) OR (eyelid laxity[Title/Abstract])) OR (floppy eyelid syndrome[Title/Abstract])) AND (OSAS[Title/Abstract])) OR (obstructive sleep apnoea syndrome[Title/Abstract])) OR (Apnoea hypopnoea syndrome[Title/Abstract])) AND (prevalence[Title/Abstract]) | 187 |
| Web of Science | ((((((TI=(floppy eyelid)) OR TI=(floppy eyelid syndrome)) OR TI=(eyelid laxity)) AND TI=(OSAS)) OR TI=(obstructive sleep apnoea syndrome)) OR TI=(apnoea hypopneoa syndrome)) AND TI=(prevalence) | 174 |
| Cochrane Library | (Floppy eyelid syndrome):ti,ab,kw OR (Lax Eyelid syndrome):ti,ab,kw AND (obstructive sleep apnoea syndrome):ti,ab,kw OR (Apnoea hypopnoea syndrome):ti,ab,kw AND (prevalence):ti,ab,kw | 135 |
| Google Scholar | prevalence "Floppy eyelid syndrome" | 1040 |
