# Supplementary material for: Lax eyelid condition (LEC) and floppy eyelid syndrome (FES) prevalence in obstructive sleep apnea syndrome (OSA) patients: a systematic review and meta-analysis
Source: Graefes Arch Clin Exp Ophthalmol. 2022 Nov 16;261(6):1505–14. doi: 10.1007/s00417-022-05890-5 (PMC10198907; doi:10.1007/s00417-022-05890-5)
Supplement: Supplementary file 2 — S1. General Features of the included studies. (Abbreviations: SD - Standard deviation -; Geo - geographical localization of the study -; RoB - Risk of Bias -; LEC – Lax Eyelid Condition -; FES – Floppy Eyelid Syndrome -; NA – Not Available -.) (DOCX 15 kb) [file 417_2022_5890_MOESM2_ESM.docx]

| Author | Year | Included patients | Female | Investigated Condition | Age +/- SD | Geo | RoB |
| --- | --- | --- | --- | --- | --- | --- | --- |
| Cristescu TR, et al | 2020 | 65 | NA | LEC and FES in OSAS | NA | Europe | High |
| Sward M, et al | 2018 | 37 | NA | LEC in OSAS | NA | North America | Moderate |
| Muniesa M, et al | 2014 | 152 | 33 | FES in OSAS | 63.66 +/- 9.01 | Europe | Moderate |
| Acar M, et al | 2013 | 280 | 83 | LEC in OSAS | 47.30 +/- 3.08 | Asia | Moderate |
| Muniesa M, et al | 2013 | 114 | 29 | LEC and FES in OSAS | 55.1 +/- 9.41 | Europe | Low |
| Chambe J, et al | 2011 | 127 | 51 | LEC and FES in OSAS | 56.6 +/- 2.70 | Europe | Moderate |
| Beis PG, et al | 2012 | 135 | NA | LEC and FES in OSAS | 49.58 +/- 12.54 | Europe | Moderate |
| Kadyan A, et al | 2009 | 115 | 23 | LEC in OSAS | 55.75 +/- 10.97 | Europe | Moderate |
| Karger RA et al | 2006 | 59 | 23 | LEC and FES in OSAS | 62.7 +/- 15.02 | North America | Moderate |
| Mojon DS, et al | 1999 | 72 | 12 | FES in OSAS | 53.57 +/- 2.45 | Europe | Low |
| Robert PY, et al | 1997 | 69 | 19 | LEC in OSAS | 57.4 +/- 13.5 | Europe | Moderate |
|  | | | | | | | |
