# Supplementary material for: Lax eyelid condition (LEC) and floppy eyelid syndrome (FES) prevalence in obstructive sleep apnea syndrome (OSA) patients: a systematic review and meta-analysis
Source: Graefes Arch Clin Exp Ophthalmol. 2022 Nov 16;261(6):1505–14. doi: 10.1007/s00417-022-05890-5 (PMC10198907; doi:10.1007/s00417-022-05890-5)
Supplement: Supplementary file 3 — S2. Risk of bias assessment according to the Joanna Briggs Institute Prevalence Critical Appraisal Tool. (a. Was the sample frame appropriate to address the target population?; b. Were study participants recruited in an appropriate way?; c. Was the sample size adequate?; d. Were the study subjects and setting described in detail?; e. Was data analysis conducted with sufficient coverage of the identified sample?; f. Were valid methods used for the identification of the condition?; g. Was the condition measured in a standard, reliable way for all participants?; h. Was there appropriate statistical analysis?; i. Was the response rate adequate, and if not, was the low response rate managed appropriately?) (DOCX 15 kb) [file 417_2022_5890_MOESM3_ESM.docx]

| **Author** | **Sample frame^a^** | **Randomization^b^** | **Sample size^c^** | **Description^d^** | **Coverage bias^e^** | **Outcome measures^f^** | **Data collection^g^** | **Statistical analysis^h^** | **Response rate^i^** | **RoB** |
| --- | --- | --- | --- | --- | --- | --- | --- | --- | --- | --- |
| Cristescu TR, et al | Yes | NA | Unclear | Unclear | No | Yes | Unclear | Unclear | Yes | High |
| Sward M, et al | Yes | NA | Yes | No | Yes | Unclear | Yes | Yes | Yes | Moderate |
| Muniesa M, et al | Yes | NA | Unclear | Yes | Yes | Yes | Yes | Yees | Yes | Moderate |
| Acar M, et al | Yes | NA | Yes | Yes | Yes | No | Yes | Yes | Yes | Moderate |
| Muniesa M, et al | Yes | NA | Yes | Yes | Yes | Yes | Yes | Yes | Yes | Low |
| Beis PG, et al | Yes | NA | Unclear | Yes | Yes | Unclear | Yes | Yes | Yes | Moderate |
| Chambe J, et al | Yes | NA | Unclear | Yes | Yes | Yes | Yes | Yes | Yes | Moderate |
| Kadyan A, et al | Yes | NA | Unclear | Yes | Yes | Yes | Yes | Yes | Yes | Moderate |
| Karger RA et al | Yes | NA | Unclear | Yes | Yes | Yes | Yes | Yes | Yes | Moderate |
| Mojon DS, et al | Yes | NA | Yes | Yes | Yes | Yes | Yes | Yes | Yes | Low |
| Robert PY, et al | Yes | NA | Unclear | Unclear | Yes | Unclear | Yes | Yes | Yes | Moderate |
