# Supplementary figures and images for: Lax eyelid condition (LEC) and floppy eyelid syndrome (FES) prevalence in obstructive sleep apnea syndrome (OSA) patients: a systematic review and meta-analysis
Source: Graefes Arch Clin Exp Ophthalmol. 2022 Nov 16;261(6):1505–14. doi: 10.1007/s00417-022-05890-5 (PMC10198907; doi:10.1007/s00417-022-05890-5)

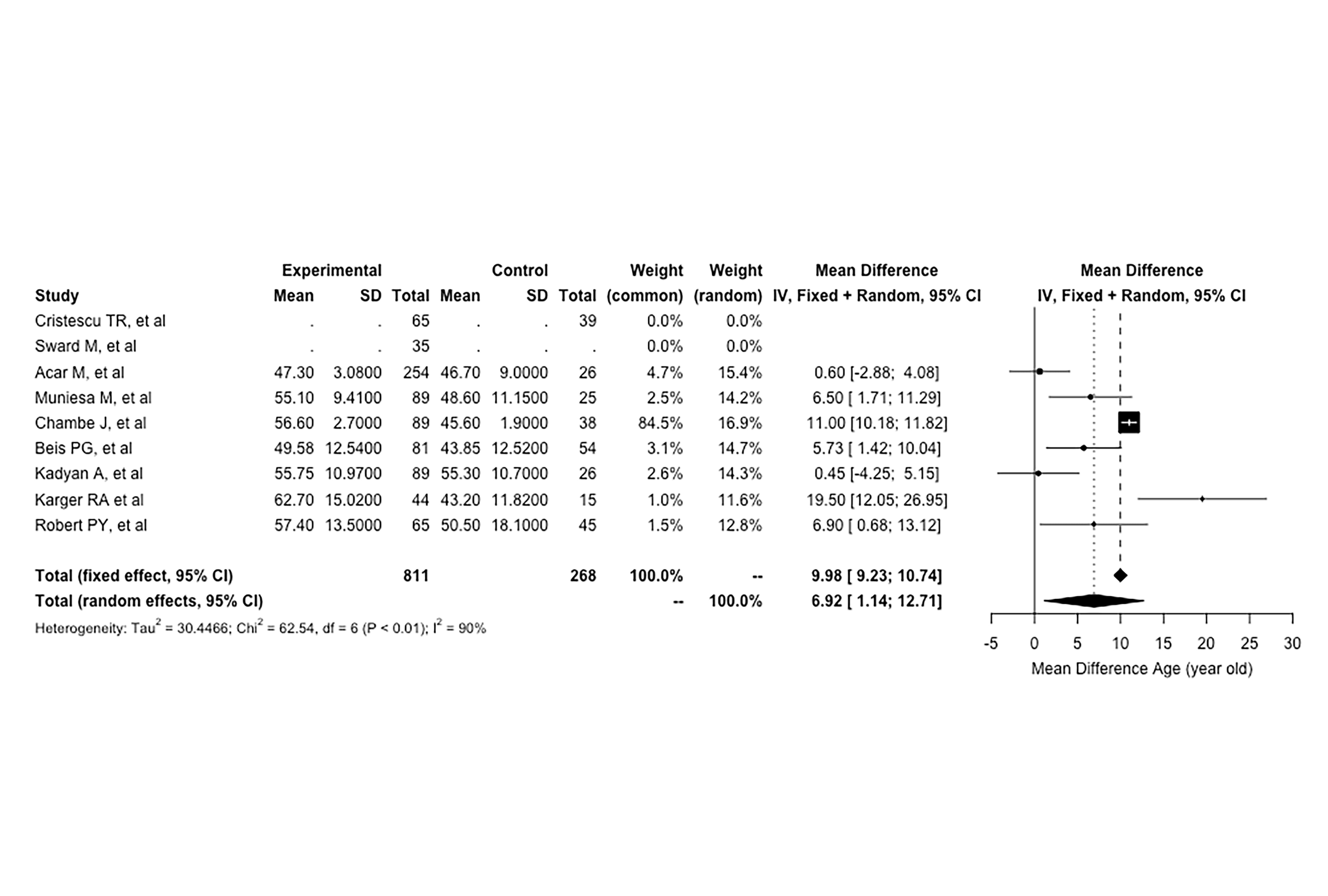

Supplement: Supplementary file 4 — S3. Forest plot showing mean difference of age between the experimental and the control group in the included studies analyzing the prevalence of lax eyelid condition. (PNG 948 kb) [file 417_2022_5890_Fig6_ESM.png]

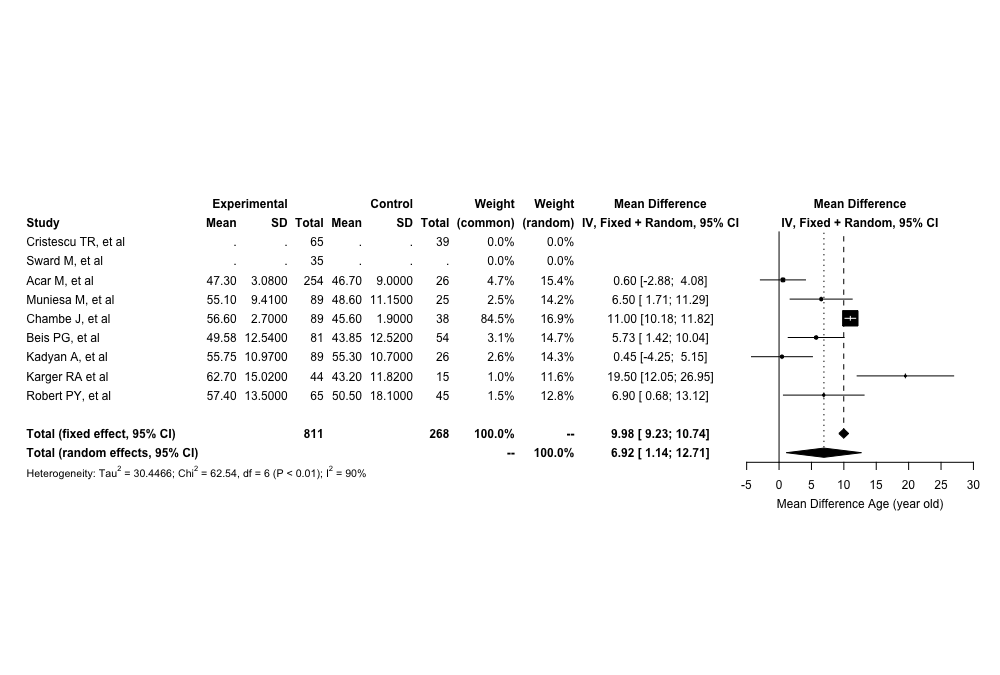

Supplement: Supplementary file 5 — High Resolution Image (TIFF 2639 kb) [file 417_2022_5890_MOESM4_ESM.tiff]

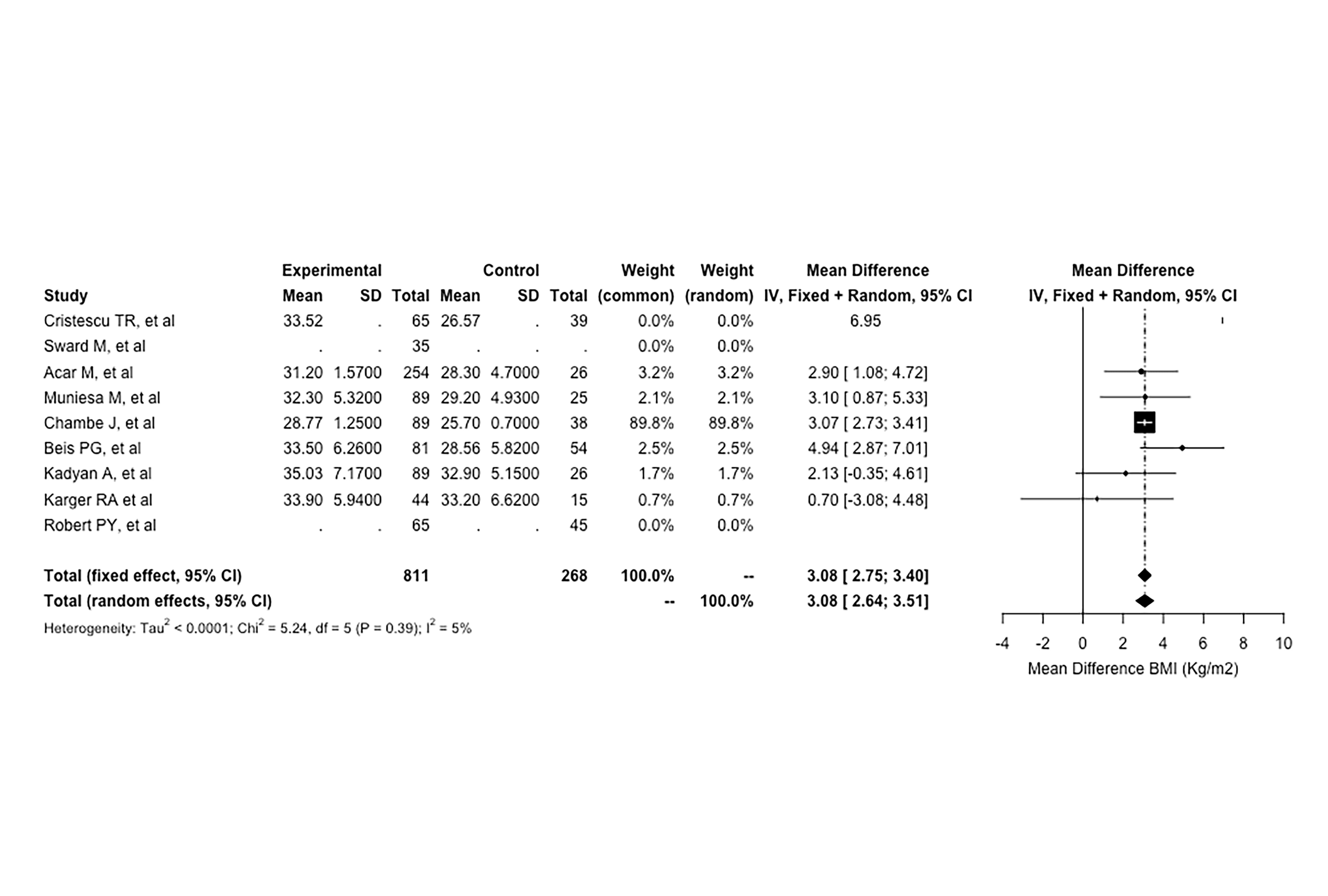

Supplement: Supplementary file 6 — S4. Forest plot showing mean difference of body mass index between the experimental and the control group in the included studies analyzing the prevalence of lax eyelid condition. (PNG 902 kb) [file 417_2022_5890_Fig7_ESM.png]

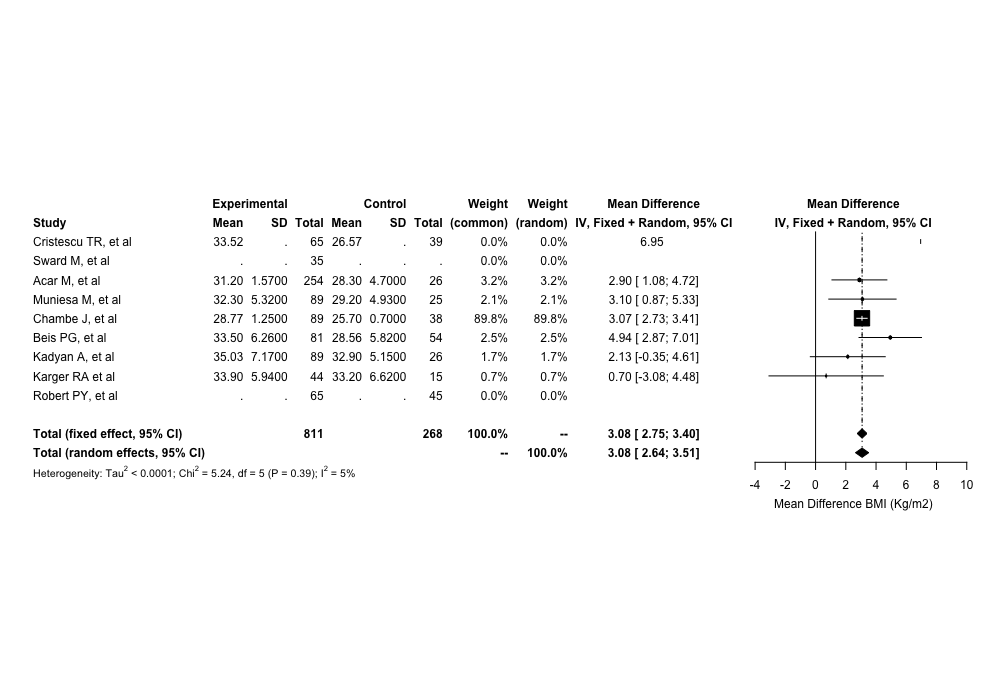

Supplement: Supplementary file 7 — High Resolution Image (TIFF 2639 kb) [file 417_2022_5890_MOESM5_ESM.tiff]

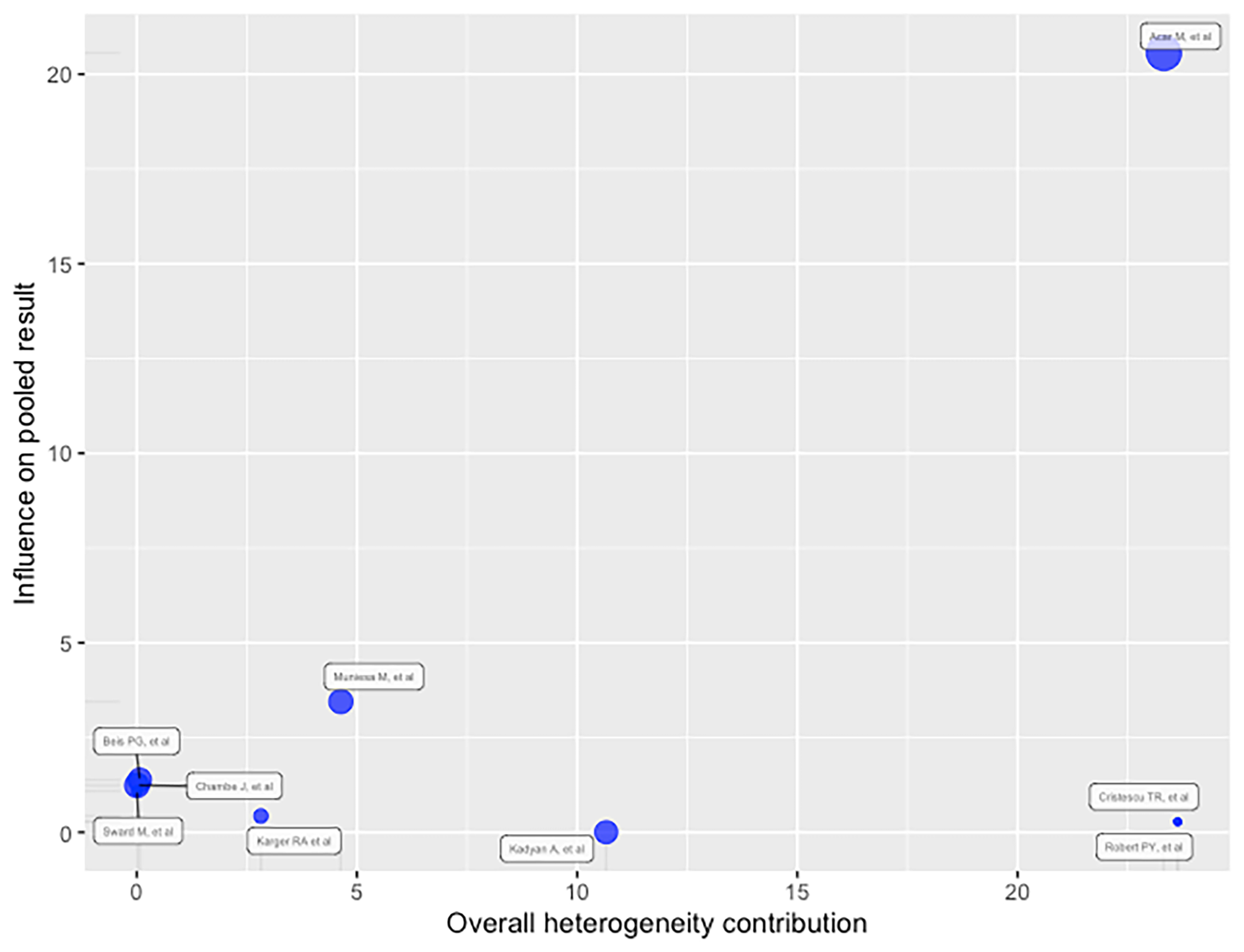

Supplement: Supplementary file 8 — S5. Baujat plot of the studies analyzing the prevalence rate of lax eyelid condition. (PNG 329 kb) [file 417_2022_5890_Fig8_ESM.png]

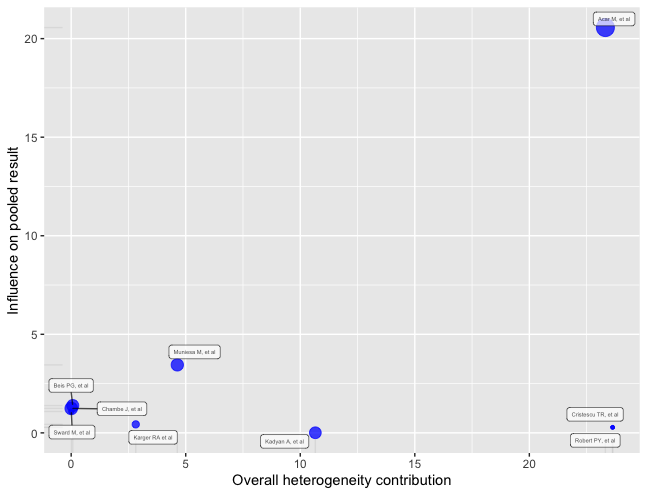

Supplement: Supplementary file 9 — High Resolution Image (TIFF 1253 kb) [file 417_2022_5890_MOESM6_ESM.tiff]

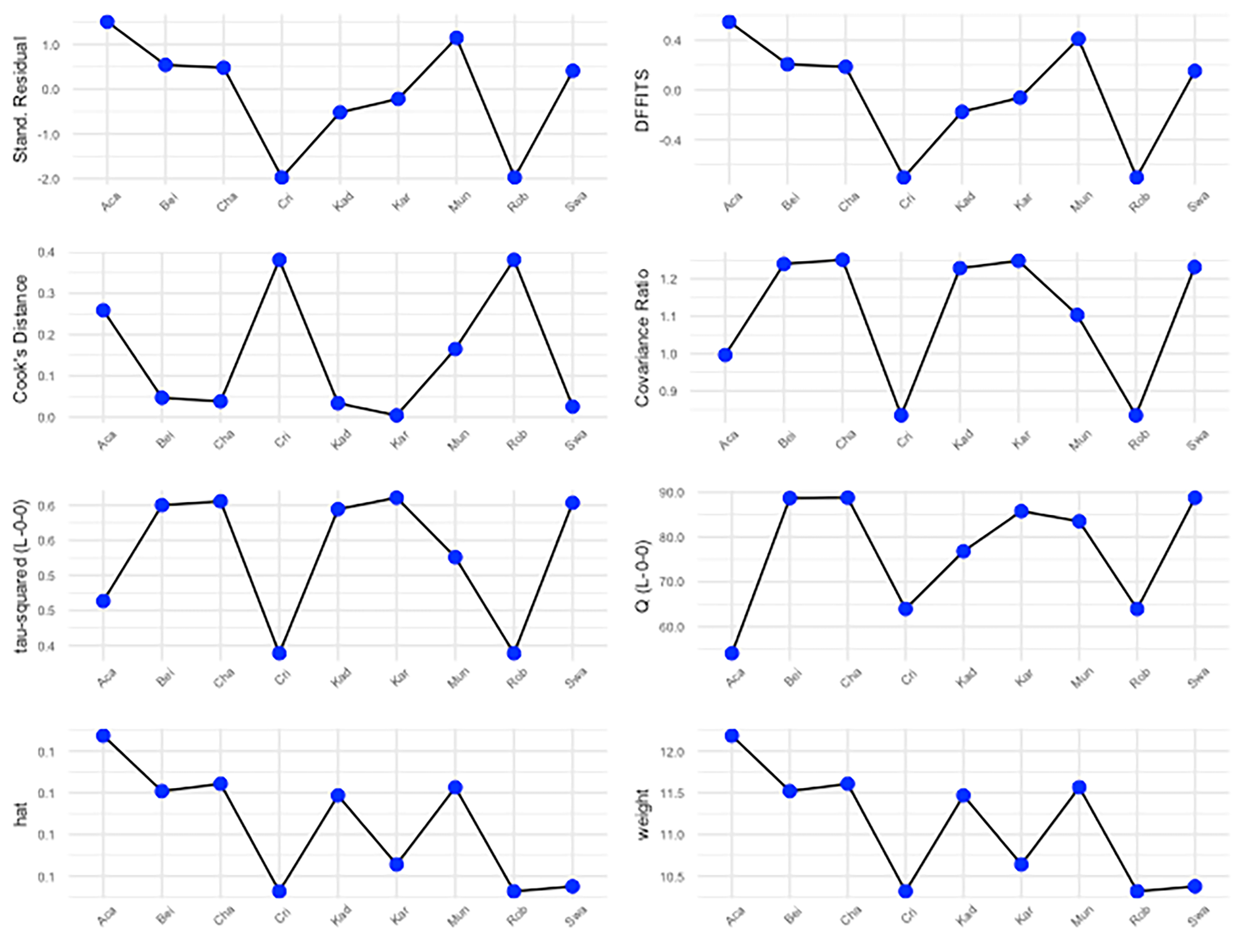

Supplement: Supplementary file 10 — S6. Influence analysis of the studies analyzing the prevalence rate of lax eyelid condition. (PNG 939 kb) [file 417_2022_5890_Fig9_ESM.png]

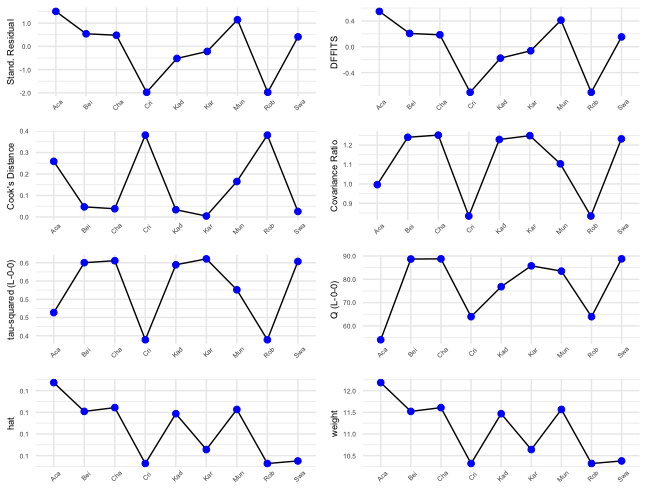

Supplement: Supplementary file 11 — High Resolution Image (TIFF 1253 kb) [file 417_2022_5890_MOESM7_ESM.tiff]

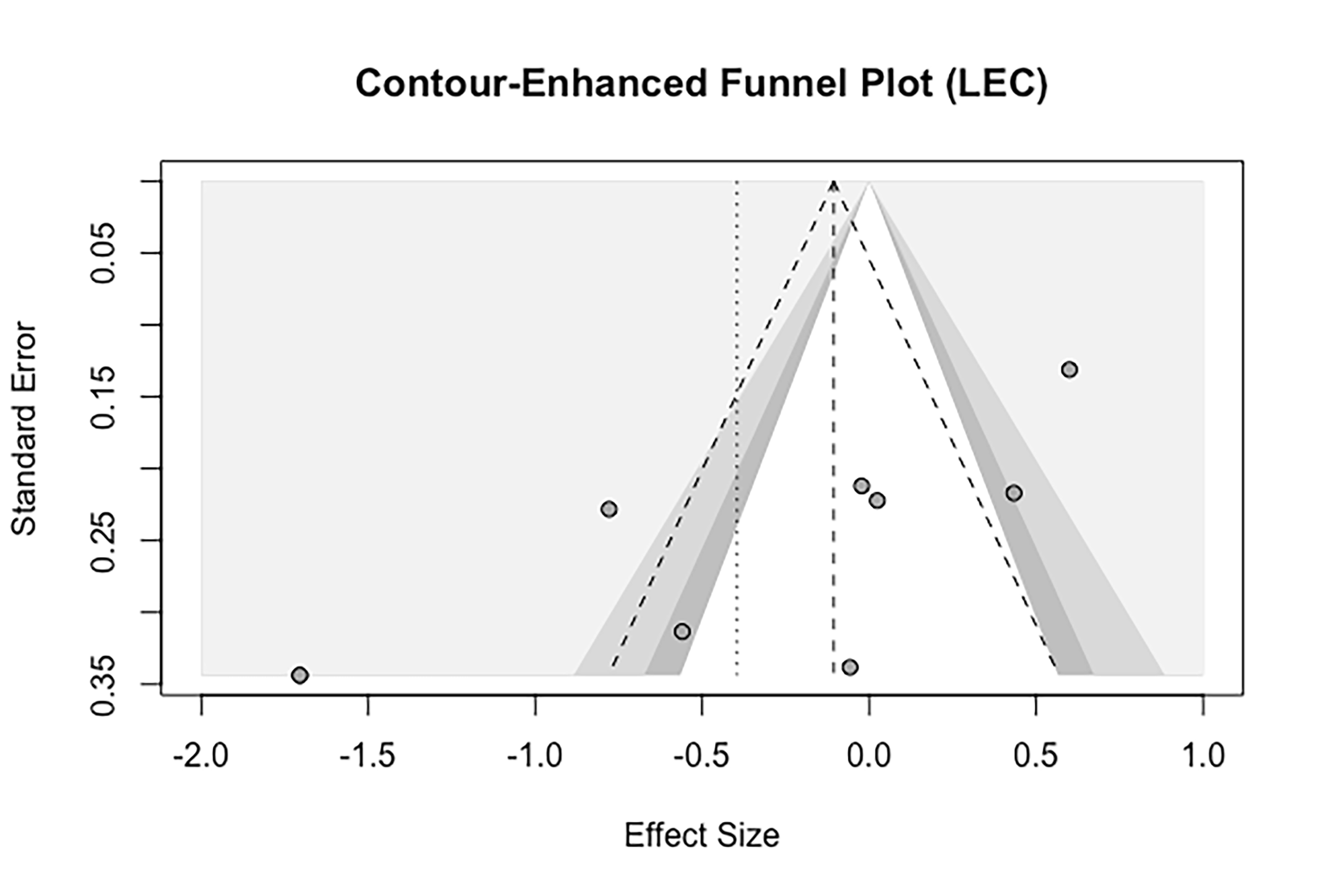

Supplement: Supplementary file 13 — S8. Contour enhanced funnel plot of the studies analyzing the prevalence rate of lax eyelid condition. (LEC: Lax Eyelid Condition) (PNG 362 kb) [file 417_2022_5890_Fig10_ESM.png]

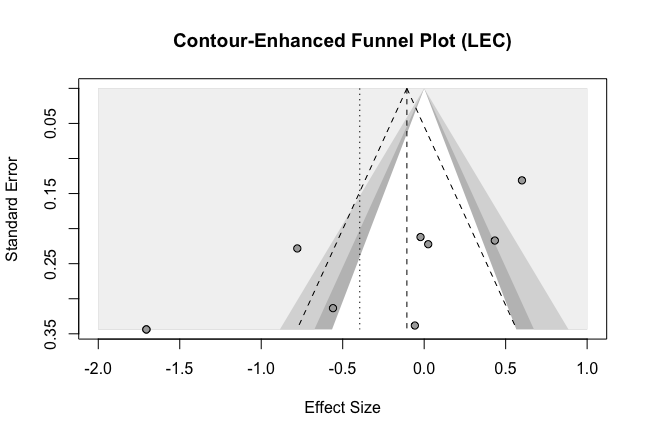

Supplement: Supplementary file 14 — High Resolution Image (TIFF 1106 kb) [file 417_2022_5890_MOESM9_ESM.tiff]

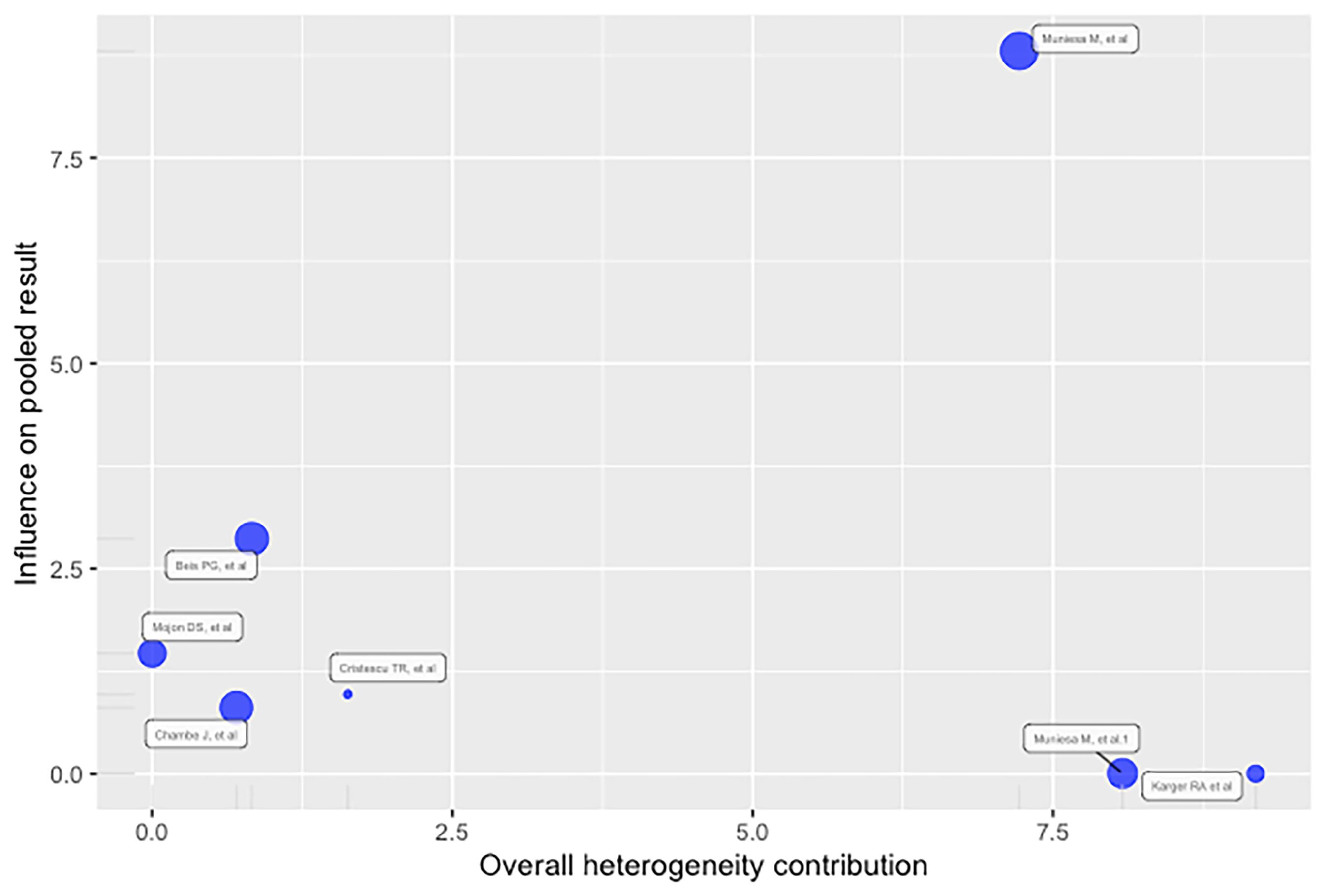

Supplement: Supplementary file 15 — S9. Baujat plot of the studies analyzing the prevalence rate of floppy eyelid syndrome. (PNG 310 kb) [file 417_2022_5890_Fig11_ESM.png]

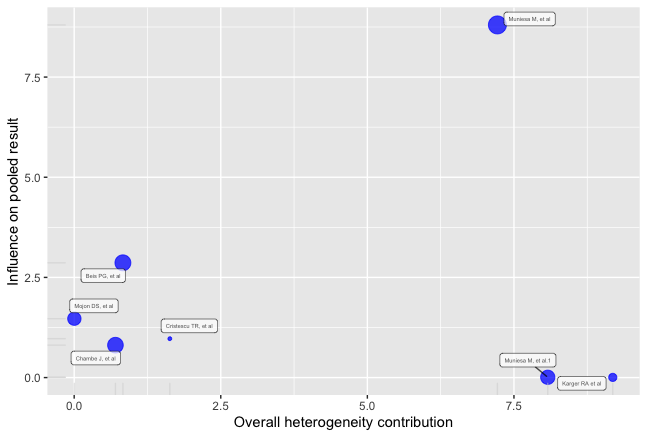

Supplement: Supplementary file 16 — High Resolution Image (TIFF 1106 kb) [file 417_2022_5890_MOESM10_ESM.tiff]

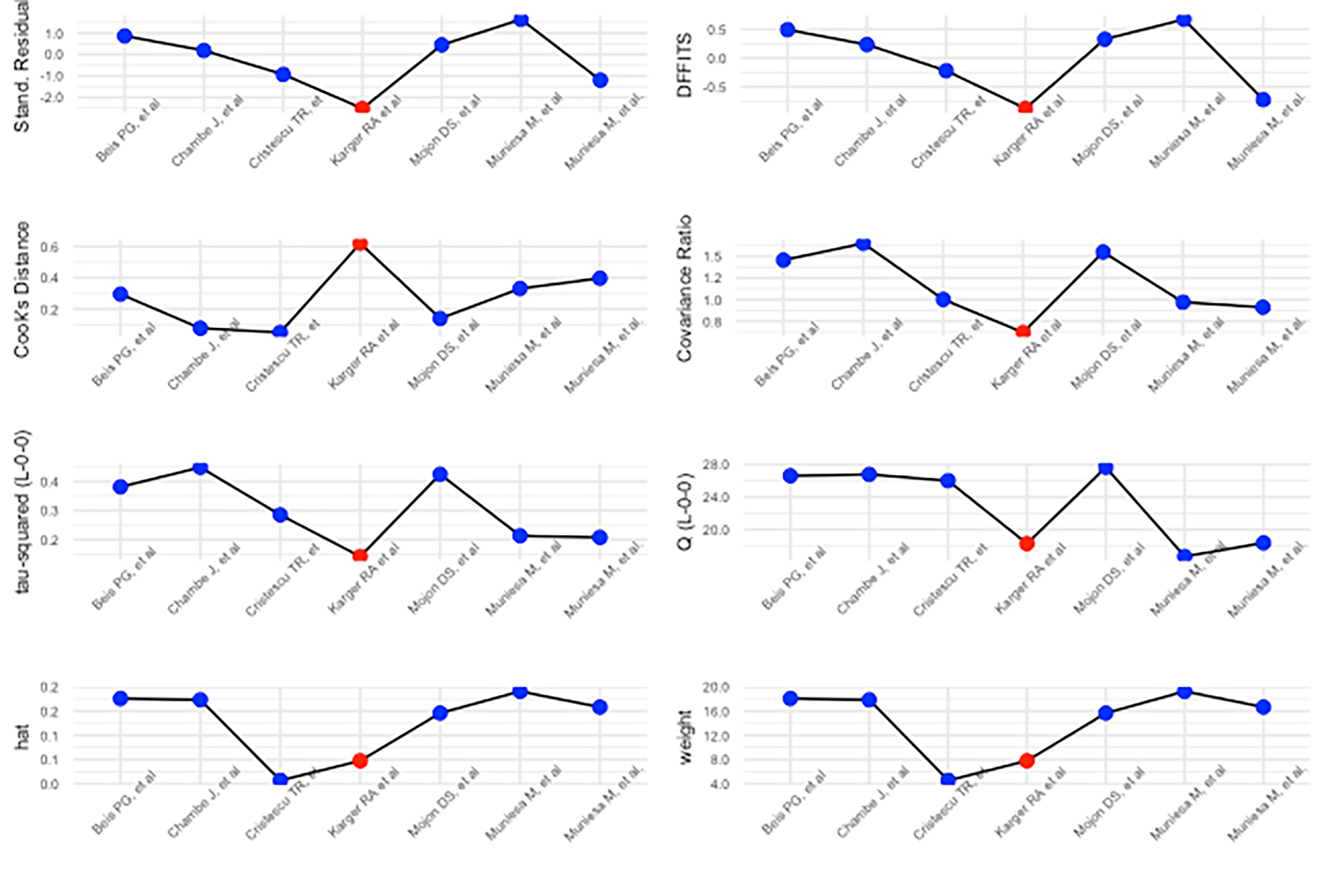

Supplement: Supplementary file 17 — S10. Influence analysis of the studies analyzing the prevalence rate of floppy eyelid syndrome. (PNG 883 kb) [file 417_2022_5890_Fig12_ESM.png]

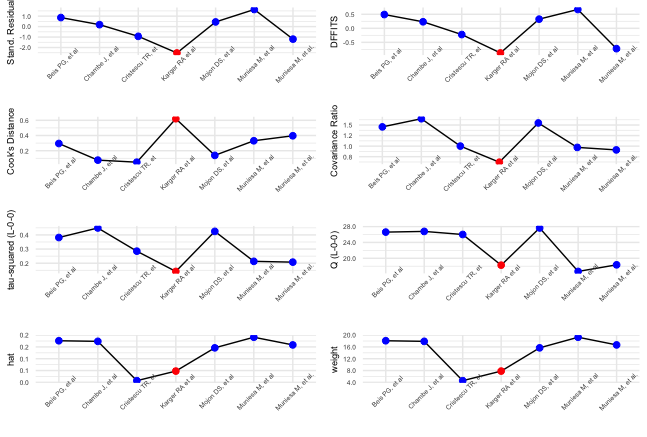

Supplement: Supplementary file 18 — High Resolution Image (TIFF 1106 kb) [file 417_2022_5890_MOESM11_ESM.tiff]

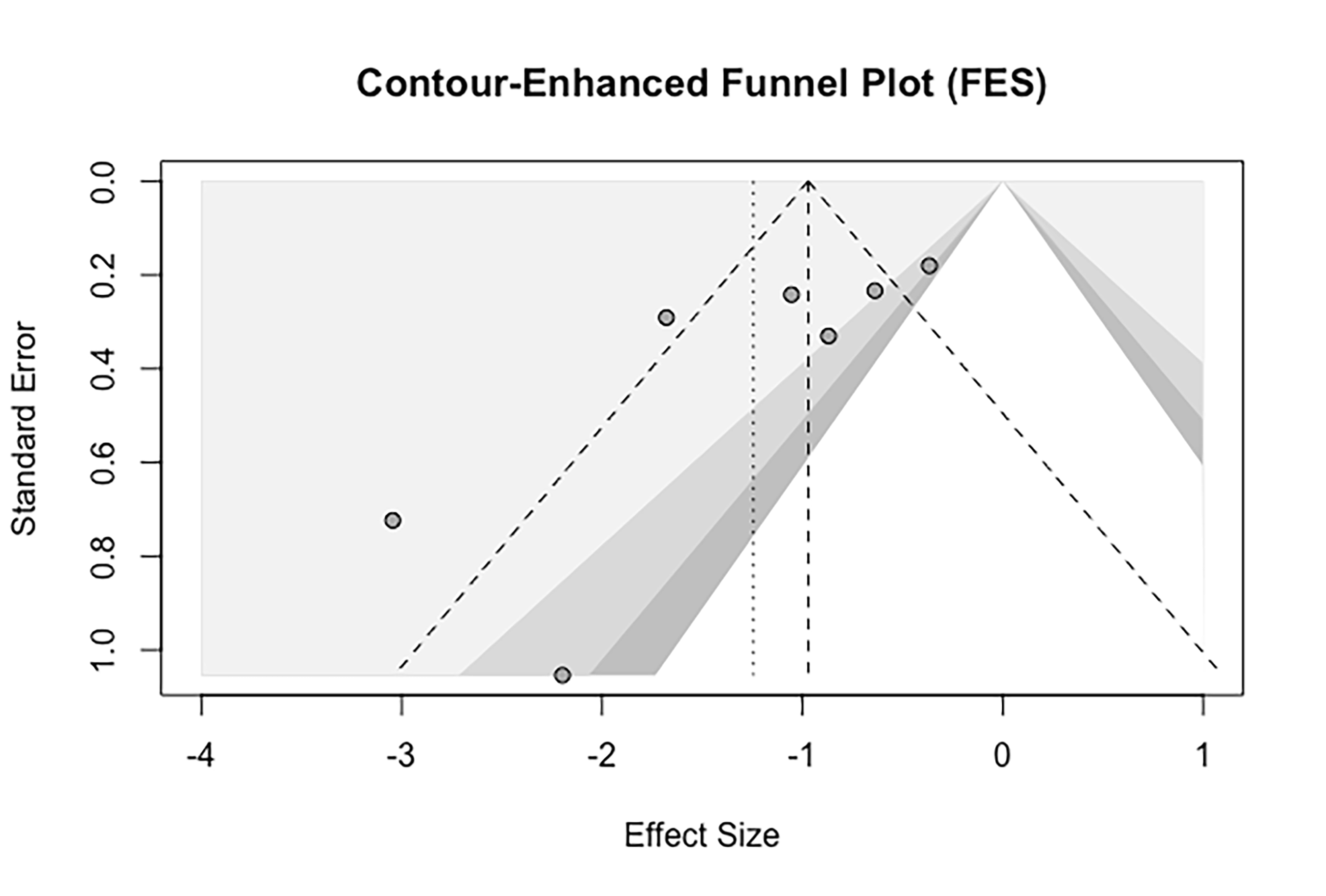

Supplement: Supplementary file 20 — S12. Contour enhanced funnel plot of the studies analyzing the prevalence rate of floppy eyelid syndrome. (FES: Floppy Eyelid Syndrome) (PNG 358 kb) [file 417_2022_5890_Fig13_ESM.png]

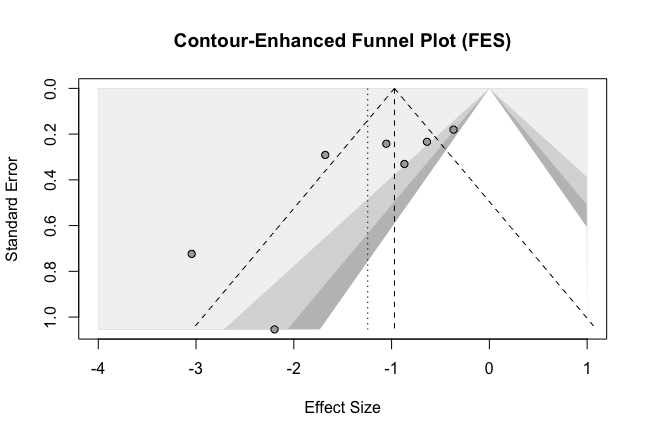

Supplement: Supplementary file 21 — High Resolution Image (TIFF 1106 kb) [file 417_2022_5890_MOESM13_ESM.tiff]
