# Supplementary material for: Lax eyelid condition (LEC) and floppy eyelid syndrome (FES) prevalence in obstructive sleep apnea syndrome (OSA) patients: a systematic review and meta-analysis
Source: Graefes Arch Clin Exp Ophthalmol. 2022 Nov 16;261(6):1505–14. doi: 10.1007/s00417-022-05890-5 (PMC10198907; doi:10.1007/s00417-022-05890-5)
Supplement: Supplementary file 12 — S7. Sensitivity analysis conducted in accordance with the run influence analysis. (LEC: Lax Eyelid Condition; CI: Confidence interval; OSA: obstructive sleep apnea syndrome) (DOCX 12 kb) [file 417_2022_5890_MOESM8_ESM.docx]

| **Analysis** | **LEC prevalence in OSAS** | **95%CI** | **t^2^** | **I^2^ (95%CI)** | **Prediction Interval** |
| --- | --- | --- | --- | --- | --- |
| **Pooled Analysis** | 40.24 | 28.57 - 53.14 | 0.5654 | 91.0% (85.2% - 94.5%) | 9.27 - 81.62 |
| **Analysis post-removal of outliers** | 46.32 | 38.11 - 54.75 | 0.11 | 71.4% (33.6% - 87.7%) | 23.11 - 71.25 |
