# Supplementary material for: Lax eyelid condition (LEC) and floppy eyelid syndrome (FES) prevalence in obstructive sleep apnea syndrome (OSA) patients: a systematic review and meta-analysis
Source: Graefes Arch Clin Exp Ophthalmol. 2022 Nov 16;261(6):1505–14. doi: 10.1007/s00417-022-05890-5 (PMC10198907; doi:10.1007/s00417-022-05890-5)
Supplement: Supplementary file 19 — S11. Sensitivity analysis conducted in accordance with the run influence analysis. (FES: Floppy Eyelid Syndrome; CI: Confidence interval; OSA: obstructive sleep apnea syndrome) (DOCX 14 kb) [file 417_2022_5890_MOESM12_ESM.docx]

| **Analysis** | **LEC prevalence in OSAS** | **95%CI** | **t^2^** | **I^2^ (95%CI)** | **Prediction Interval** |
| --- | --- | --- | --- | --- | --- |
| **Pooled Analysis** | 22.38 | 13.79 - 34.18 | 0.4518 | 78.3% (55.1% - 89.5%) | 4.16 - 65.67 |
| **Analysis post-removal of the most influential case** | 27.58 | 20.19 – 36.45 | 0.1567 | 72.6% (37.0% - 88.1%) | 9.90 - 56.89 |
